# Abnormal Fhit expression is an independent poor prognostic factor for cervical cancer

**DOI:** 10.1038/sj.bjc.6600892

**Published:** 2003-04-15

**Authors:** S Takizawa, S Nakagawa, K Nakagawa, T Yasugi, T Fujii, K Kugu, T Yano, H Yoshikawa, Y Taketani

**Affiliations:** 1 Department of Obstetrics and Gynecology, Graduate School of Medicine, University of Tokyo, Hongo 7-3-1, Bunkyo-ku, Tokyo 113-8655, Japan; 2 Department of Radiology, Graduate School of Medicine, University of Tokyo, Hongo 7-3-1, Bunkyo-ku, Tokyo 113-8655, Japan; 3 Department of Obstetrics and Gynecology, Institute of Clinical Medicine, University of Tsukuba, 1-1-1 Tennodai, Tsukuba City, Ibaragi 305-8575, Japan

**Keywords:** cervical cancer, Fhit expression, HPV

## Abstract

We analysed the expression of the fragile histidine triad (FHIT) gene in cervical cancer to evaluate its clinical relevance in relation to human papillomavirus (HPV) infection. A total of 73 women with cervical cancer of stage Ib or more advanced (67 squamous cell carcionomas, four adenocarcinomas, two adenosquamous carcinomas) were examined for Fhit expression by immunohistochemistry. They were further analysed for the presence of HPV and its subtype. Abnormal expression of Fhit (absent or reduced Fhit expression) was observed in 52 cases (71.2%). The high-risk HPV DNAs for cervical cancer, including type 16, 18, 31, 33, 51, 52, 58, 68, were identified in 63 cases (86%). The abnormal Fhit expression was not related to the clinicopathological factors including histology, tumour stage, and HPV type. Notably, the 5-year survival of patients showing the abnormal Fhit expression was significantly poorer than those showing normal Fhit expression (64 versus 87%, *P*=0.035). Interestingly, the mean age of the patients with the abnormal Fhit expression was significantly less than those with the normal Fhit expression (51.6 versus 58.7 years of age, *P*=0.027, student's *t*-test). These data imply that the aberrant Fhit expression could be a poor prognostic factor independent of HPV. In the light of a high incidence of abnormal Fhit expression in younger patients and HPV as a key player in cervical carcinogenesis, abnormal Fhit expression may accelerate carcinogenesis in concert with HPV.

Human papillomavirus (HPV) infection is a major causal factor for the carcinogenesis of cervical cancer and it is thought to be an early event of the multistep development of cervical cancer. Human papillomavirus infection, however, is identified in about 10% of normal women without any abnormality of cervical cytology, with a very small portion of women with HPV infection developing cervical cancer, suggesting that mechanisms other than HPV infection might be involved in cervical carcinogenesis.

The aberrant transcripts of the fragile histidine triad (FHIT) gene have been identified in some cancers including cervical cancer and CINs (Negrini *et al*, 1996; Ohta *et al*, 1996; Sozzi *et al*, 1996; Virgilio *et al*, 1996; Greenspan *et al*, 1997; Hendricks *et al*, 1997; Hadaczek *et al*, 1998; Nakagawa *et al*, 1999; Segawa *et al*, 1999). Higher incidences of the aberrant FHIT expression in invasive cervical cancer and high-grade CIN than in low-grade CIN or the normal cervical epithelium suggest that the alteration of FHIT expression is involved in cervical carcinogenesis (Nakagawa *et al*, 1999). To date, two studies described the abnormal Fhit expression. For instance, Krivak *et al* (2001) reported that the abnormal Fhit was an independent poor prognostic factor for stage II – III patients with cervical cancer of squamous type. In contrast, Helland *et al* (2000) described that the abnormal Fhit was associated with poor prognosis, but not an independent poor prognostic factor by analysing Fhit expression in cervical cancers for all stages and histological type. These conflicting results give rise to the question whether or not the alteration of the FHIT gene is really the causal event in the cancer development. To address this, we analysed Fhit protein expression in primary cervical cancer tissues using the immunohistochemical-staining technique with special reference to an association of the abnormal Fhit expression with clinical characters.

## MATERIALS AND METHODS

### Specimens

A total of 73 cases were enrolled in this study, including 67 squamous cell cancers, four adenocarcinomas, and two adenosquamous cancers. The distribution of the tumour stages is as follows: stage Ib; 20 cases; stage II; 36 cases; stage III; 13 cases; stage IV; four cases. The mean age of the cases is 54.8 years (28–82 years). We also examined cervical tissues from women undergoing hysterectomy due to benign gynaecological conditions. All the subjects gave informed consent for study and had treatment in the university of Tokyo hospital in 1987 – 1997.

### Analysis of Fhit expression

Each slide, after being deparaffinised and hydrated, was placed in a container and covered with 0.01 M sodium citrate buffer (pH 6.0) and heated in a microwave oven (500 W) four times for 5 min each. After washing in deionised water, endogenous peroxidase was blocked with 3% hydrogen peroxide for 5 min. The tissue sections were further blocked with 10% normal pig serum (NPS) (Kohjin Bio, Japan) in phosphate-buffered saline (PBS) for 10 min at room temperature to saturate nonspecific binding sites. Then, they were incubated with diluted primary antibody, anti-FHIT (ZYMED, San Francisco, CA, USA) at 1 : 200 in PBS overnight at 4°C. For negative control, the slides were incubated with 10% NPS. The slides were rinsed in PBS, and incubated with biotinylated F(ab′)_2_ fragment of swine anti-rabbit immunoglobulins (DAKO, Tokyo, Japan) at 1 : 500 in PBS for 30 min at room temperature. The slides were rinsed in PBS, incubated with peroxidase-conjugated streptavidin (DAKO, Tokyo, Japan) at 1 : 500 in PBS for 30 min at room temperature, followed by rinsing in PBS, incubation with DAB (20 mg 3,3′-diaminobenzidine, tetrahydrochloride) for 5 min at room temperature, and counterstaining with haematoxylin for 1 min.

The degree of Fhit expression was evaluated semiquantitatively measuring both intensity (no expression or very weak, 1; moderate, 2; strong positive, 3) and extent (per cent of positive staining; <10%, 1, 10–50%, 2, >50%, 3). The score of the intensity was multiplied by the score of the extent to give the total score for the immunohistochemical staining of Fhit. The total score of 4–9 was judged as normal based on observations of noncancerous tissues of the uterine cervix.

### Detection and typing of HPV

PCR for the L1 region was carried out using the consensus L1 primers L1C1 (5′-CGTAAACGTTTTCCCTATTTTTTT-3′) (1 *μ*M), L1C2 (5′-TACCTAAATACTCTGTATTG-3′) (0.5 *μ*M), and L1C2M (5′-TACCCTAAATACCCTATATTG-3′) (0.5 *μ*M). Other conditions of the PCR were as described elsewhere (Yoshikawa *et al*, 1991). Briefly, the PCR protocol was 40 cycles, each cycle consisting of 1.5 min denaturation (95°C), 1.5 min annealing (48°C), and 2 min extension (70°C). One-tenth of the reaction was electrophoresed on a 4% agarose gel and stained with ethidium bromide. Human papillomavirus types were identified on the basis of the restriction fragment length polymorphisms (RFLP). The PCR-based assay (L1-PCR) can type at least 26 registered genital HPVs including type 6, 11, 16, 18, 30, 31, 33, 34, 35, 39, 42, 43, 44, 45, 51, 52, 53, 54, 55, 56, 59, 61, 66, 68, and 70. This PCR system can detect 0.01 pg of HPV DNA for all these types except for HPV DNA of types 34, 42, and 55, of which 0.1 *μ*g DNA is minimally needed for detection. We used *β*-actin gene amplification to rule out false-negative results.

### Statistical analysis

Survival of patients was analysed by the Kaplan–Meier technique. Comparison of mean age was done by Student's *t*-test. Other statistical analysis methods conducted in this study were *χ*^2^ test and multivariate analysis based on the stratified Cox proportional hazards model.

## RESULTS

**Figure 1:**
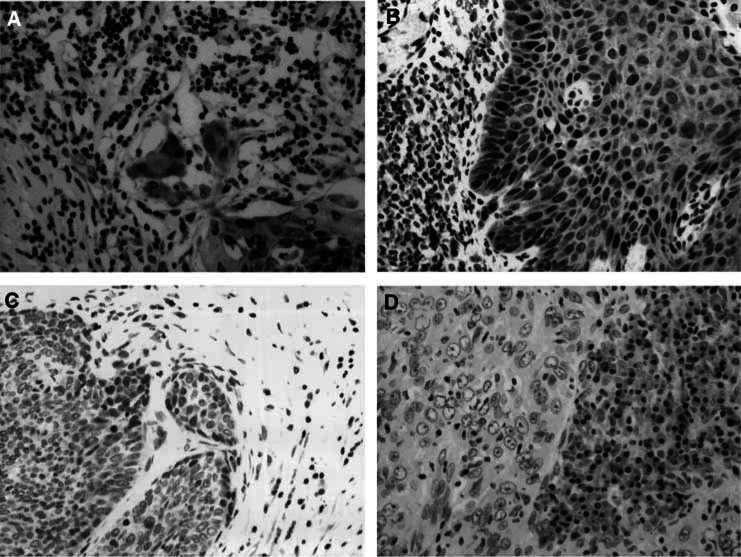
Immunohistochemical staining of Fhit protein in cervical cancer. (**A**, **B**) Normal Fhit expression in cervical cancer. The Fhit expression scores were 6 (A) and 9 (B). (**C**) Absent Fhit expression in cervical cancer. Fhit expression in the cervical cancer tissue was negative (score 0). Note that Fhit is expressed normally in the normal stromal tissue. (**D**) Marked reduced Fhit expression in cervical cancer. The Fhit expression score in cancer tissue was 3.

**Figure 2:**
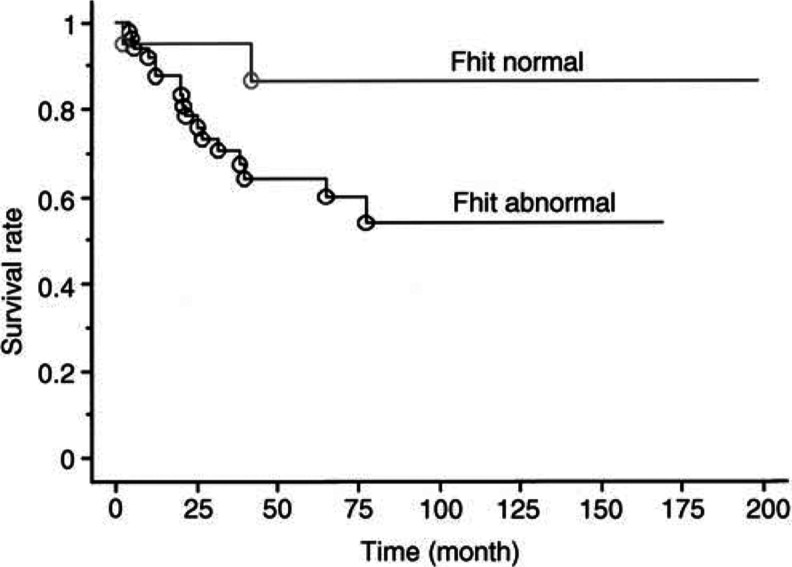
Survival of cervical cancer patients with normal and abnormal Fhit expression.

The intensity of Fhit expression was judged based on a three-tiered scale comparing Fhit expression between cancers and normal tissues, that is, the uterine cervix of unaffected women or histologically normal tissues adjacent to cancers. Fhit was expressed diffusely in the cytoplasm of epithelial and stromal cells in the normal tissues (Figure 1A and B

). Of the 73 cases with cervical cancer, 52 cases (71.2%) showed abnormal expression of Fhit protein. Abnormal Fhit expression, that is, marked reduction or an absence of Fhit protein expression, is shown in Figure 1C and D, respectively. The survival of the patients was compared between the normal Fhit expression and the abnormal Fhit expression using the Kaplan–Meier technique. The 5-year survival of the abnormal group was significantly poorer than the normal group (64 versus 87%, *P*=0.035, Figure 2

). The mean age of the abnormal group was significantly younger than the normal group (51.6 years versus 58.7 years, *P*=0.027). In patients at the age of 50 years or older, the abnormal Fhit expression was detected in 29 out of 46 (63%). In contrast, 23 out of 27 cases (85%) had the abnormal Fhit expression in patients lesser than 50 years of age, the difference reaching statistical significance (*P*<0.05, Table 1

**Table 1:** Fhit expression and clinicopathological characters of 73 cervical cancers in Japanese women

	**Total**	**Abnormal Fhit expression**	**Normal Fhit expression**	***P***
Number of patients	73	52	21	
				
*Age*				
≤50	27	23(85%)	4(15%)	
>50	46	29(63%)	17(37%)	0.043
*Histology*				
Squamous cell cancer	67	48(72%)	19(28%)	
Adenocarcinoma	4	1(25%)	3(75%)	
Adenosquamous cancer	2	2(100%)	0(0%)	0.289
				
*Stage*	20	15(75%)	5(25%)	
Ib	36	24(67%)	12(33%)	
II	13	10(77%)	3(23%)	
III	4	3(75%)	1(25%)	0.528
IV				
				
*HPV types*				
16	28	20(71%)	8(29%)	
18	9	4(44%)	5(56%)	
Others	26	19(73%)	7(27%)	
Negative	10	9(90%)	1(10%)	0.201

). We further analysed the relation between the Fhit expression and the mean age at onset by dividing patients into four groups according to the Fhit expression score (Table 2

**Table 2:** Fhit expression and age at onset of 73 cervical cancers in Japanese women

	**Fhit expression (score)**				
	**Negative (0)**	**Low (1 – 3)**	**Medium (4 – 6)**	**High (7 – 9)**	**Total**
Number of cases	3	40	25	5	73
Mean age at onset	42.7	52.3	57.0	67.0	54.5

). The mean age at onset showed a tendency of increase along with the rise of the Fhit expression score. Then we looked at whether Fhit expression is related to the clinical staging. The abnormal Fhit expression was observed in 15 out of the 20 cases with stage Ib (75%), in 24 out of the 36 cases with stage II (67%), in 10 out of the 13 cases with stage III (77%), and in three out of the four cases with stage IV (75%). There was no significant statistical difference in the rates of the abnormal Fhit expression among different clinical stages. We next analysed the relation between the abnormal Fhit expression and HPV infection. The high-risk HPVs including HPV type 16, 18, 31, 33, 51, 52, 58, and 68 were identified in 63 cases (86%). The abnormal Fhit expression was observed in 20 out of the 28 cases positive for HPV 16 (71%), in four out of the nine cases positive for HPV 18 (44%), in 19 out of the 26 cases infected with the other types of HPV (type 31, 33, 51, 52, 58, and 68) (73%), and in nine out of the 10 cases without HPV infection (90%). The abnormal Fhit expression was not related to the detection of HPV or its type. The multivariate analysis with respect to the clinical stage and the age at onset revealed that the abnormal Fhit expression remains as an independent poor prognostic factor (*P*<0.05).

## DISCUSSION

At present, only a small body of existing literature has described the clinical implication of normal Fhit expression in cervical cancer. The present data seem to be in keeping with a previous report by Krivak *et al* (2001) who claimed that abnormal Fhit expression was a poor prognostic factor in patients whose clinical stages were restricted to stage II or III. Here, we extended this finding such that abnormal Fhit expression is an independent poor prognostic factor for all clinical stages inclusive. In contrast, the data by Helland *et al* (2000) failed to demonstrate the abnormal Fhit expression as an independent poor prognostic factor unrelated to HPV infection. This disparity may be explained, in part, by the difference of the breakdown of histology or HPV subtype examined.

It was reported that Fhit protein expression was markedly reduced or absent in 67 out of 95 (71%) invasive cancer tissues, 17 out of 33 (52%), and eight out of 38 (21%) in high-grade CIN tissues associated or unassociated with invasive cancer, respectively (Connolly *et al*, 2000). Considering this and the findings from our laboratory, the alteration of FHIT expression could occur at the stage of high-grade CIN and could be, along with HPV, an important molecular event pertinent to the progression of invasive cervical cancer.

Human papillomavirus infection is thought to serve as an initiator for cervical cancer. It follows that HPV infection occurs well before the alteration of Fhit expression. On the other hand, the present study demonstrated the abnormal Fhit expression as a poor prognostic factor independent of HPV infection and its subtype. Besides, our finding of a higher incidence of the abnormal Fhit expression in relatively younger women with cervical cancer is noteworthy. If we assume that the age of HPV infection is essentially the same in cervical cancer patients with or without abnormal Fhit expression, the above finding could be interpreted to suggest a role for the abnormal Fhit expression as an accelerator of carcinogenesis.

At present, we have shown that clinical courses of cervical cancer are different depending on HPV subtype (Nakagawa *et al*, 1996). Here, we propose a new determinant for prognosis of cervical cancer. The abnormal Fhit expression may accelerate the neoplastic process initiated by HPV infection. The question of whether or not the abnormal Fhit expression is involved in the carcinogenesis of cervical cancer by a mechanism similar to HPV is still open.
